# The First Defined Null Allele of the Notch Regulator, a Suppressor of Deltex: Uncovering Its Novel Roles in *Drosophila melanogaster* Oogenesis

**DOI:** 10.3390/biom14050522

**Published:** 2024-04-26

**Authors:** Marian B. Wilkin, Rory Whiteford, Tanveer Akbar, Samira Hosseini-Alghaderi, Raluca Revici, Ann-Marie Carbery, Martin Baron

**Affiliations:** Manchester Academic Health Science Centre, School of Biological Sciences, University of Manchester, Michael Smith Building and Oxford Rd., Manchester M13 9PT, UK

**Keywords:** suppressor of deltex, ubiquitin ligase, notch, drosophila, development, oogenesis, stem cells, tissue homeostasis, endocytosis

## Abstract

Suppressor of deltex (*Su(dx)*) is a *Drosophila melanogaster* member of the NEDD4 family of the HECT domain E3 ubiquitin ligases. *Su(dx)* acts as a regulator of Notch endocytic trafficking, promoting Notch lysosomal degradation and the down-regulation of both ligand-dependent and ligand-independent signalling, the latter involving trafficking through the endocytic pathway and activation of the endo/lysosomal membrane. Mutations of *Su(dx)* result in developmental phenotypes in the *Drosophila* wing that reflect increased Notch signalling, leading to gaps in the specification of the wing veins, and *Su(dx)* functions to provide the developmental robustness of Notch activity to environmental temperature shifts. The full developmental functions of *Su(dx)* are unclear; however, this is due to a lack of a clearly defined null allele. Here we report the first defined null mutation of *Su(dx)*, generated by P-element excision, which removes the complete open reading frame. We show that the mutation is recessive-viable, with the Notch gain of function phenotypes affecting wing vein and leg development. We further uncover new roles for *Su(dx)* in *Drosophila* oogenesis, where it regulates interfollicular stalk formation, egg chamber separation and germline cyst enwrapment by the follicle stem cells. Interestingly, while the null allele exhibited a gain in Notch activity during oogenesis, the previously described *Su(dx)^SP^* allele, which carries a seven amino acid in-frame deletion, displayed a Notch loss of function phenotypes and an increase in follicle stem cell turnover. This is despite both alleles displaying similar Notch gain of function in wing development. We attribute this unexpected context-dependent outcome of *Su(dx)^sp^* being due to the partial retention of function by the intact C2 and WW domain regions of the protein. Our results extend our understanding of the developmental role of *Su(dx)* in the tissue renewal and homeostasis of the *Drosophila* ovary and illustrate the importance of examining an allelic series of mutations to fully understand developmental functions.

## 1. Introduction

Notch signalling has many fundamental and diverse roles during both development and adult tissue homeostasis [1]. Its core pathway is initiated by ligand-induced proteolytic cleavage that removes most of the extracellular domain (ECD), followed by intramembrane processing that releases the intracellular domain (ICD) [2]. The latter becomes a component in a transcription factor complex involving the transcription factor Suppressor of Hairless and coactivator protein Mastermind. In this active ICD-bound form, the complex binds to Su(H) DNA binding motifs and regulates the expression of target genes such as the E(spl) complex [3,4]. Despite the relative simplicity of the core pathway mechanism, Notch is involved in a wide variety of cell fate decisions and different tissue patterning contexts, such as lateral inhibition, boundary formation and asymmetric cell fate decisions. In order for Notch signalling to be deployed in this wide range of different contexts, a number of regulatory systems have evolved to tune the amplitude and duration of Notch activity, according to different physiological inputs and developmental patterning requirements. For example, carbohydrate modifications of the ECD determine its affinities for different ligands and ICD modifications, such as phosphorylation and ubiquitination, which are associated with proteosomal degradation, intracellular trafficking, lysosomal degradation and ligand-independent activation mechanisms [5,6,7]. Suppressor of deltex (*Su(dx)*) is a HECT domain E3 ubiquitin ligase of the Nedd4 family whose origin in evolution predates the origin of core Notch pathway components [8]. In yeast, for example, the yeast homologues RSP5 and Pub1 are involved in environmental sensing mechanisms that regulate the trafficking and activity of nutrient transporters [9,10]. In humans, Nedd4-2 has been linked to the trafficking of a Na channel involved in salt balance and blood pressure regulation [11]. *Su(dx)* was originally linked to Notch by the positional cloning of gene mutations that dominantly suppress the mutant phenotypes of another ubiquitin ligase regulator of Notch called Deltex [12], which itself acts to promote ligand-independent signal activation [13,14]. Other Nedd4 family members have been linked to Notch regulation across a number of metazoan species [15,16,17,18].

In a wild-type setting, mutations of *Su(dx)* alone cause wing vein gap phenotypes symptomatic of gain-of-function Notch activity [12]. The mutant phenotype is temperature-sensitive, with increased Notch activity observed at high temperatures. It was found that *Su(dx)* acts with Deltex in a robustness module that stabilised the Notch signalling across the physiological temperature range of the fly through a balance of temperature-dependent fluxes affecting the relative contributions of ligand-dependent and independent signals [14]. By acting in a network of trafficking routes, both *Su(dx)* and Dx can act positively or negatively on Notch, sometimes acting antagonistically to each other, and sometimes acting together in the same direction. For example, double homozygous alleles of *dx* and *Su(dx)* produce extra leg joints, an outcome of increased Notch activity, but the mutants mutually suppress each other’s phenotypes in wing development above 18 °C, and combine to produce Notch loss of function phenotypes below 18 °C [14]. *Su(dx)* is more effective at down-regulating Notch at higher temperatures due to the temperature-dependent activation of its ubiquitin ligase function. Other developmental functions of *Su(dx)* are revealed by the genetic interactions between *Su(dx)* alleles and Notch pathway mutants. For example, *Su(dx)* mutants suppress their wing notching phenotypes due to the loss of one copy of the *Notch* gene, or the homozygous *nd^1^* allele of *Notch* [12]. Existing mutant alleles of *Su(dx)* are either uncharacterised molecularly, carry a small in-frame deletion in the HECT domain (*Su(dx)^sp^*), or are truncation mutants which leave open the possibility of the expression of a part of the protein [8,12]. To examine the full requirement for *Su(dx)*, it is necessary to generate a defined null allele in which the complete open reading frame is deleted. In this study, we generated the first defined null allele, *Su(dx)^JD^*, using imprecise p-element excision [19]. We show that, in the wing and leg tissues, the null phenotype is essentially similar to the phenotypes reported for existing *Su(dx)* alleles, i.e., it demonstrates similar temperature-dependent outcomes and genetic interactions with *dx* and *Notch* alleles. We also identifynew phenotypes arising in adult female oogenesis. Interestingly, the observed phenotypes in the ovary differ markedly between *Su(dx)^JD^* and the other *Su(dx)* alleles tested. The former display phenotypes characteristic of a Notch signal gain of function while combinations of the other alleles displayed reduced stalk length, incomplete separation of successive egg chambers and compound egg chambers, characteristic of a Notch loss of function. The null allele further reveals a novel role for *Su(dx)* in regulating the cell extensions of follicle progenitor cells that cross the germarium between successive germline cysts, and whose misregulation results in split cysts. We also attribute this phenotype to a Notch gain of function. This work thus identifies new roles for *Su(dx)* in the adult homeostasis of the ovary and highlights how the complexity of genotype/phenotype links requires the consideration of a range of mutant alleles.

## 2. Materials and Methods

### 2.1. Drosophila melanogaster Strains

*Su(dx)^JD^* was generated from the imprecise excision [19,20] of P-element insertion line P*{GSV1}Su(dx)EP-735* (Bloomington Stock Center, Bloomington, IN, USA) by crossing w[*]; wg[Sp-1]/CyO; and ry[506] Sb[1] P{ry[+t7.2] = Delta2-3}99B/TM6B, Tb[+] (Bloomington, IN, USA) and screening the excision lines lacking a *w*+ marker by polymerase chain reaction (PCR) and sequencing using the flanking primers TCGAATGATAGGCGAAATGAGC and ACGAAACAATACACGCGTCG. The additional *Drosophila* mutant lines used were *Su(dx)^sp^*, *Su(dx)^56^* [8,12], the *Notch* null allele *N^55e11^* (Bloomington, IN, USA), the *deltex* null allele *dx^152^* [21] and a genomic rescue construct *Su(dx)*GR [14]. For the signalling assays a Notch reporter element (NRE)-driven expression of GFP was utilised. For the ovaries: w1118; P{NRE-EGFP.S}5A #30728, w1118; P{NRE-EGFP.S}1 (Bloomington, IN, USA), and w[1118]; P{w[+mC] = 10XStat92E-GFP}2. For wing discs the N^sf-GFP^ reporter, which drives a nuclear localised PEST, destabilised GFP under the control of the NRE [22]. To express NRNAi we used y[1] v[1]; P{y[+t7.7] v[+t1.8] = TRiP.HMS00001}attP2 pVALIUM20 Notch (Bloomington, IN, USA). UAS-Notch ICD was a gift from Spyros Artavanis-Tsakonas (Harvard Medical School, Boston, MA, USA). The endogenously EYFP-tagged endosomal marker fly lines used were TI{TI}Rab4[EYFP], TI{TI}Rab5[EYFP] and TI{TI}Rab7[EYFP] (Bloomington, IN, USA). The wild type was *y^1^*, *w^1^* (Bloomington, IN, USA).

### 2.2. Generation of Mitotic Clones

To enable mitotic clone generation, *Su(dx)^sp^* and *Su(dx)^JD^* alleles were recombined with frt40A (Bloomington, IN, USA). For positive marked clones, frt40, *Su(dx)* lines were crossed to hsflp1, uas cd8gfp:tubgal80, frt40A; actgal4 (Bloomington). For the follicle stem cell turnover assay, using negatively marked *Su(dx)^sp^*, *Su(dx)^JD^* and WT control clones, the following stocks were generated: hsflp^1^/+;*Su(dx)*frt40A/P{Ubi-GFP(S65T)nls}2L P{neoFRT}40A and hsflp^1^/+;frt40A/P{Ubi-GFP(S65T)nls}2L P{neoFRT}40A, using component stocks obtained from Bloomington. One-day-old adult flies were heat-shocked for 60 min at 40 °C and then reared for minimum of 1 week at 25 °C to ensure that any clones generated in the dividing follicle cells were cleared from the germarium and egg chambers and that the clones scored, up to stage 5 egg chambers inclusive, originated in the stem cells.

For the examination of the stem cell processes in positively marked clones, the following genotypes were generated, using components obtained from Bloomington stock centre. For WT clones: P{ry[+t7.2] = hsFLP}1, y[1] w[*] P{w[+mC] = UAS-mCD8::GFP.L}Ptp4E[LL4]/+; P{w[+mC] = tubP-GAL80}LL10, P{ry[+t7.2] = neoFRT}40A/frt40A; actgal4/+. For *Su(dx)^JD^* clones: P{ry[+t7.2] = hsFLP}1, y[1] w[*] P{w[+mC] = UAS-mCD8::GFP.L}Ptp4E[LL4]/+; P{w[+mC] = tubP-GAL80}LL10, P{ry[+t7.2] = neoFRT}40A/*Su(dx)*nullfrt40A; P{w[+mC] = tubP-GAL4}LL7/+. For *N* null clones: P{w[+mC] = tubP-GAL80}LL1 w[*] P{ry[+t7.2] = neoFRT}19A/N55e11 frt19A; P{w[+mC] = Act5C-GAL4}25FO1, P{w[+mC] = UAS-GFP.U}2/+; MKRS, Hsflp. For Notch ICD expression clones: hsflp1, tubgal80, frt19A/yw frt; CD8-GFP, 109-30 gal4; his 2av mRFP/UAS NICD. For Notch RNAi clones: P{w[+mC] = tubP-GAL80}LL1 w[*] P{ry[+t7.2] = neoFRT}19A/yw, frt19A; P{w[+mC] = Act5C-GAL4}25FO1, P{w[+mC] = UAS-GFP.U}2/+;MKRS, Hsflp. Clones were induced 1 day after adult female eclosion and dissected 7 days after heat shock to ensure all clones observed originated from a follicle stem cell.

### 2.3. Antibodies and Immunostaining

The primary antibodies used were Goat anti-GFP (Abcam, Cambridge, UK, used 1:250), mouse anti-Fasciclin III (Isotype IgG2A, Developmental Studies Hybridoma Bank, University of Iowa, USA used 1:40), mouse anti-Discs Large (Isotype IgG1, Developmental Studies Hybridoma Bank, University of Iowa, used 1:40) and Rabbit anti-*Su(dx)* [23] (used 1/500). The latter was first preabsorbed against crushed adult *Su(dx)^JD^* tissue for 1 week at 4 °C, in 0.1% tween to remove background staining. Secondary antibodies were obtained from Jackson ImmunoResearch Laboratories, Inc. Cambridge, UK, and used 1:500. Ovaries were dissected in ice-cold PBS and then fixed in 1 mL of 4% formaldehyde for 20 min at room temperature. After fixation, ovarioles were washed for 3 × 5 min in 0.1% PBS-Tw20. Ovarioles were then incubated with the primary antibodies in PBS-Tw20 at the given dilution, overnight at 4 °C. Antibodies were removed and washed for 6 × 5 min in 0.1% PBS-Tw20 at RT. Ovarioles were then incubated with the appropriate secondary antibody in PBS-Tw for 2 to 4 h at 4 °C in the dark. Tissue preparations were again washed for 6 × 5 min in 0.1%PBS-Tw20 at RT. If Actin was to be visualized then, following secondary antibody staining, ovarioles were incubated with 0.5% Alexa 647-Phalloidin (Thermo Fisher, Waltham, MA, USA) in PBT-Tw for 1 h. Preparations were washed for 5 × 5 min in 0.1%PBS-Tw20 before mounting in 4′.6-dianidino-2-phenylindole (DAPI) Vectashield mounting medium (Vector Laboratories). Ovarioles were examined using a Zeiss Axioskop fluorescence microscope (Carl Zeiss, Oberkochen, Germany). Images were captured using a cooled Hamamatsu digital camera and processed on an Apple Macintosh G4-500 computer using Improvision Openlab II Deconvolution and Adobe Photoshop CS5.1 software Where images were deconvoluted, serial Z sections of 0.5 µm were taken through a sample. Each layer was deconvoluted using the 3 nearest neighbours above and below the section of interest.

For the scoring of signalling levels in FSCs using GFP reporter constructs, mean fluoresence intensity was calculated for a 25 pixel diameter region in a Z-section through the plane of the FSC. The background level, from a non-stained region in the same tissue, was subtracted and the result normalised to background-subtracted GFP levels of wild-type flies. For the scoring of signalling levels in wing imaginal discs, a similar procedure was used on separate regions encompassing the dorsal/ventral boundary.

### 2.4. Adult Wing/Leg Mounting

Adult wings were dissected using fine forceps and mounted on slides in Gary’s magic mounting media (Canada balsam thinned with methysalicitate, Sigma-Aldrich, St. Louis, MO, USA).

### 2.5. Whole Mount In Situ Hybridisation

Ovaries were dissected, including sheath removal, in PBS; fixed for 20 min in 4% formaldehyde; rinsed 3 × 5 min in 0.1% PBTw (tween 20); washed 2 × 5minin ethanol, in ethanol:xylene 1:1 for 60 min, in ethanol for 10 min; and then transferred to ice-cold methanol overnight at −20 °C. Ovaries were then washed 2 × 5 min and then 3 × 10 min in 0.1% PBTw, washed in 50% hybridization washing buffer (HWB; 50% formamide, 5 × SSC, 0.1% Tween-20, adjusted to pH4/5 with 1 M citric acid) and then hybridisation buffer (Hs: HWB with 0.1 mg/mL tRNA, 50 μg/mL heparin) for 10 min at RT. Ovarioles were prehybridized in HS for 1 hr at 70 °C. Ovaries were hybridized overnight at 70 °C with a digoxigenin-labelled (Boehringer Mannheim, Mannheim, Germany) 2b1a [8] antisense probe and then washed for 2 × 20 min in HS at 70 °C, for 20 min in 50% HS in PBT at 70 °C and for 3 × 20 min in PBT at RT on a rotating wheel. Ovaries were then incubated with an alkaline phosphatase-conjugated anti-digoxigenin antibody (Roche, Basel, Switzerland) in 0.1%PBTw (1:1000) at RT for 90 min, washed for 2 × 1 min and then 3 × 20 min with 0.1% PBTw, and then washed with NMTT 2 × 5 min each (NMTT; 0.1M NaCl, 50 mM MgCl_2_, 0.1M Tris pH 9.5, 0.1% Tween-20). The antibody conjugate was detected using the substrate NBT/BCIP (Boehringer Mannheim, Mannhein, Germany), and the reaction stopped by a 3 × 20 min wash with 20mM EDTA, and ovaries were mounted in 90% glycerol.

### 2.6. Scoring of Ovariole Phenotypes

To assess egg chamber production, ovarioles were stained with DAPI and the numbers of egg chambers were scored between stage 2 (the earliest stage to exit the germarium) and stage 8. Stalk length was assessed as the mean numbers of cells present in the stalks, anterior and posterior to stage four egg chambers. To define the number of mature cysts present in the germarium, ovarioles were stained with anti-FasIII and the numbers of cysts surrounded by FasIII-positive cells were counted. Split cyst egg chambers were scored by counting the number of germ line nurses and oocytes per egg chamber. To score for delays in egg chamber separation, the stage of the most recently pinched off egg chamber to exit the posterior of the germarium was assessed according to published criteria [24].

## 3. Results

### 3.1. Generation of a Defined Null Allele of the Suppressor of Deltex

**Figure 1:**
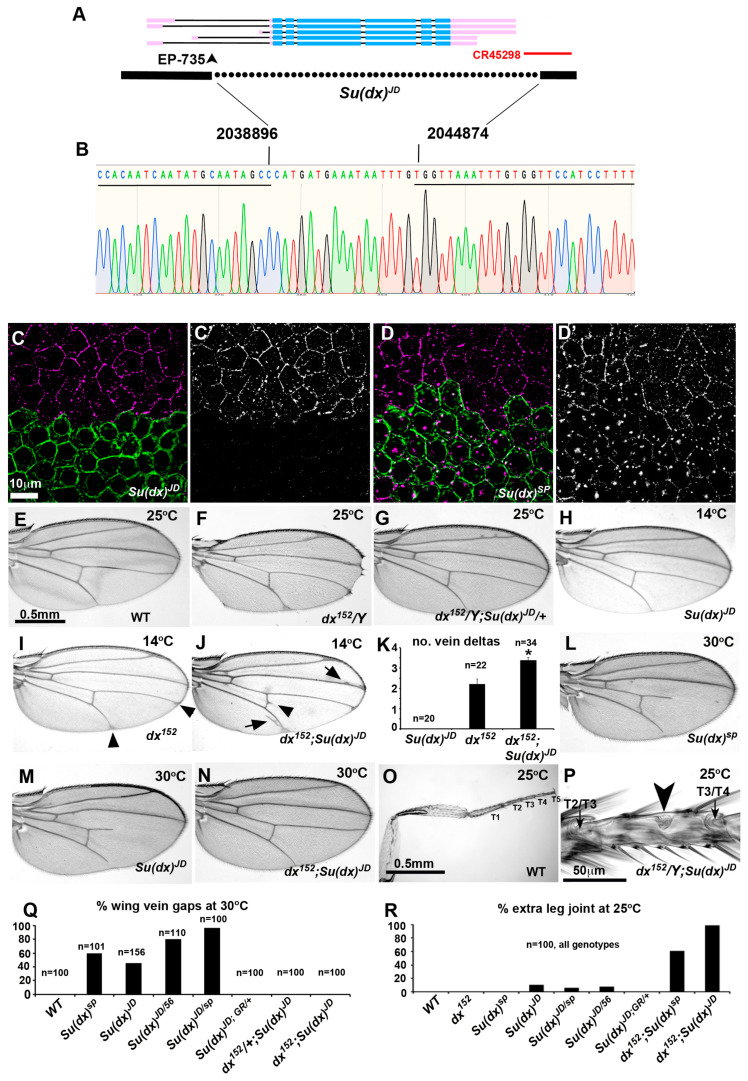
Generation of a defined *Su(dx)* null allele. (**A**) map of genomic region spanning *Su(dx)* locus, showing known splice forms. Thick lines represent exons, thin lines representintrons. Blue lines are mRNA coding regions and pink are non-coding sequences. Position of transposon used for P-element excision to create deletion is indicated with arrowhead. Dotted line below indicates region deleted in *Su(dx)^JD^* allele. Red line indicates position of non-coding RNA transcript. (**B**) Sequence across deletion breakpoint of the *Su(dx)^JD^* allele. Underlined region indicates WT sequence that lies 5′ and 3′ of deletion endpoints. Nucleotide numbers of breakpoints from 2nd chromosome genomic sequence are indicated. (**C**,**D**) Anti-*Su(dx)* immunostaining (purple) of CD8-GFP positively marked *Su(dx)^JD^* (**C**,**C’**) and *Su(dx)^sp^* (**D**,**D’**) mitotic clones in stage 8 egg chamber. Green staining indicates CD8-GFP-expressing cells, which are homozygous for *Su(dx)* mutants. *Su(dx)^JD^* mutant cells do not stain with anti-*Su(dx)* antibody but *Su(dx)^sp^* mutant cells show immunostaining in vesicular organelles and reduced staining at cell boundaries. Images show deconvolved Z-sections. (**E**) Wild-type wing. (**F**) Adult wing of *dx^152^* fly bred at 25 °C showing wing notching and vein thickening phenotype. (**G**) *Su(dx)^JD^* dominantly suppresses *dx* mutant phenotype. (**H**) *Su(dx)^JD^* mutant wing at 14 °C appears to be wild-type. (**I**) *dx^152^* wing at 14 °C displays vein delta phenotype (arrowheads). (**J**) At 14 °C, *dx^152^;Su(dx)^JD^* double mutant wings have increased frequency and severity of vein delta phenotypes and also display vein thickening (arrows) and cross-vein spurs (arrowheads). (**K**) Quantification of 14 °C vein delta phenotypes. * indicates *p* < 0.001 compared to *dx^152^* by *t*-test, error bars and SEM. (**L**,**M**) *Su(dx)^sp^* and *Su(dx)^JD^* mutant wings, from female flies bred at 30 °C, displaying wing vein gap phenotypes. (**N**) *dx^152^* mutant suppresses *Su(dx)^JD^* wing vein gap phenotype. (**O**) Wild-type male back leg. Tarsal regions 1–5 are indicated. (**P**) Tarsal region 3 of fly which is double null mutant for *Su(dx)^JD^* and *dx^152^* mutants. Arrowhead indicates ectopic joint. Arrows indicate normal joints located between adjacent T2 and T4 tarsals. (**Q**,**R**) Quantification of wing vein gap and extra leg joint and their phenotypes at 30 °C and 25 °C, respectively. Genotypes are indicted below.

To generate a null allele of *Su(dx)*, an imprecise excision of P-element EP-735A was performed and the extent of the resulting deletion confirmed by sequencing, utilising PCR primers flanking the *Su(dx)* locus (Figure 1A,B). A new, homozygous, viable null allele, *Su(dx)^JD^*, was generated, in which the complete coding sequence of the *Su(dx*) gene was removed. The loss of *Su(dx)* protein expression was confirmed by the immunostaining of mitotic clones using a previously generated antibody, which targets the WW domain region of *Su(dx)*. Endogenous *Su(dx)* was detected both at the cell–cell junctions and in punctate organelles within the cytoplasm (Figure 1C,D). Staining with compartment markers showed that *Su(dx)* was localised to endosomal-pathway-associated organelles including Rab4-, Rab5- and Rab7-positive compartments and at the cell boundaries (Supplementary Figure S1A–C). Clones of the homozygous *Su(dx)^JD^* allele showed a loss of immunolocalization compared to heterozygous regions outside of the clone (Figure 1C,C’). In contrast, *Su(dx)*’s immunofluorescence was observed in organelle compartments in clones of the *Su(dx)^sp^* allele, which carries a seven amino acid in-frame deletion in the HECT domain (Figure 1D,D’). Immunostaining of the *sp* allele was also observed at the cell boundaries, although this was reduced compared to WT. 

**Figure 2:**
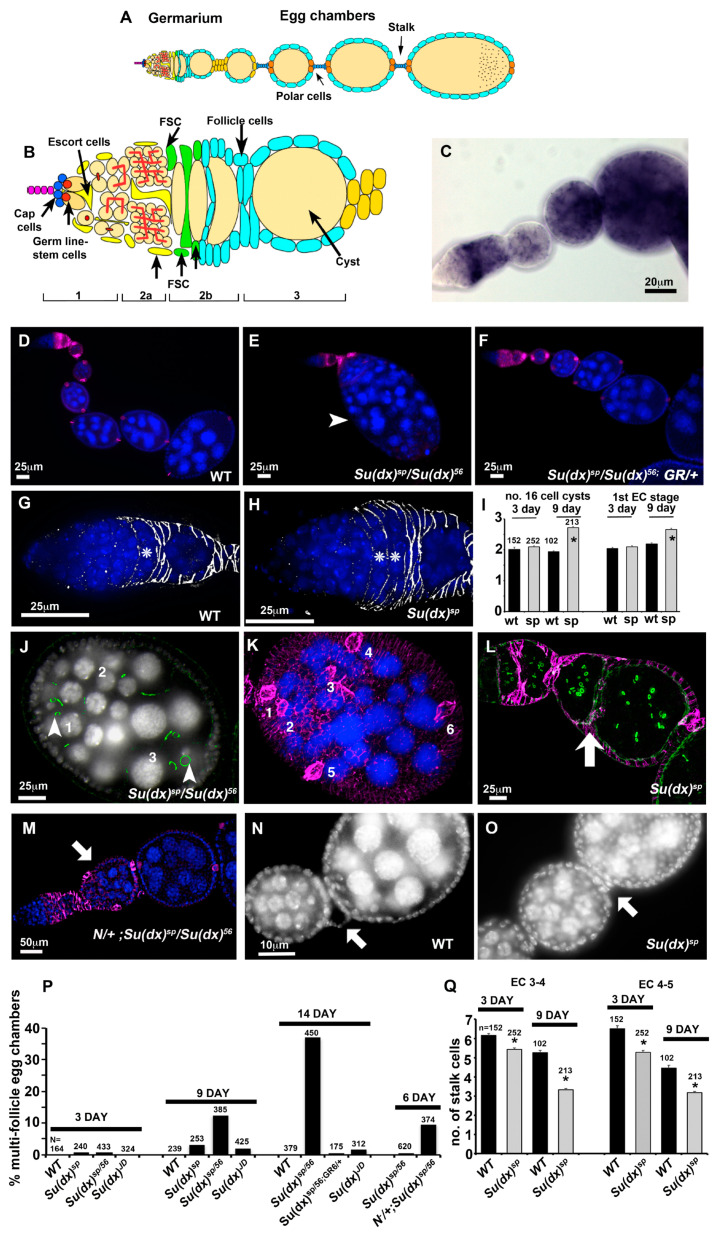
Age-dependent ovary phenotypes of *Su(dx)* mutant alleles. (**A**) Schematic diagram of ovariole. (**B**) Germarium region showing position of germline and follicle stem cells and switch in enwrapment of germline cysts from escort cells to follicle cells. (**C**) in situ mRNA expression of *Su(dx)* in ovariole. (**D**) Wild-type ovariole stained with dapi (blue) and anti-FasIII (purple). (**E**) *Su(dx)^sp^* allele fails to complement *Su(dx)^56^* allele for a compound egg chamber phenotype. A large multi-cyst egg chamber is shown in image (arrowhead). (**F**) Rescue of *Su(dx)* ovariole phenotype by a wild-type genomic rescue construct (GR). (**G**) Wild-type germarium showing single FasIII (greyscale)-enwrapped 16-cell cyst, indicated by *. (**H**) *Su(dx)^sp^* germaria have increased numbers of FasIII-enwrapped 16-cell cysts, indicated by *. (**I**) Scoring of FasIII-enwrapped cysts and 1st egg chamber stage in wild-type and *Su(dx)^sp^* mutant ovarioles at 3 days and 9 days post eclosion. * denotes *p* < 0.05 compared to wild-type ovarioles, error bars are SEM, numbers scored for each genotype are indicated. Same cohort were scored for both phenotypes. (**J**) Egg chamber of *Su(dx)^sp^/Su(dx)^56^* mutant ovariole with 3 complete germline cysts; Actin staining (phalloidin, green) indicates location of ring canals. Arrowheads indicate two out of the three oocytes present, each surrounded by 4 ring canals. (**K**) Same egg chamber as in (**J**), showing anti-FasIII staining (purple). Numbers indicate 6 pairs of strongly FasIII-expressing polar cells, consistent with the inclusion of 3 separate cysts into a single compound egg chamber. Image is a composite of merged deconvolved z-sections. (**L**) *Su(dx)^sp^* mutant ovariole showing egg chambers separated by an epithelial bilayer (arrow) but lacking an intervening stalk and polar cells; phalloidin (green) marks ring canals, and follicle cells marked with anti-FasIII (purple). (**M**) Phenotype of heterozygous *Notch* null allele combined with *Su(dx)* at 6 days post eclosion, showing compound egg chamber (arrow). (**N**) Wild-type egg chambers separated by a linear arrangement of stalk cells (arrow). (**O**) *Su(dx)^s^*^p^ ovarioles display shorter stalks containing fewer cells (arrow). (**P**) Scoring of multi-follicle egg chamber phenotypes. Age post eclosion and numbers scored are indicated. (**Q**) Quantification of stalk length phenotypes, comparing stalk cell numbers anterior and posterior to stage 4 egg chambers. The same cohort of ovarioles is scored in (**P**,**Q**) as in (**I**). * denotes *p* < 0.05 compared with WT of same age, error bars SEM.

We compared the phenotypes of *Su(dx)^JD^* with those previously described for existing *Su(dx)* alleles. As with other alleles, *Su(dx)^JD^* acted as a dominant suppressor of the *dx* mutant phenotype (Figure 1E–G), suppressing the wing margin and vein thickening phenotypes, and also dominantly suppressing the wing margin phenotype of *notchoid^1^* (*nd^1^*), a hypomorphic allele of Notch. This combination also produced a wing vein gap phenotype that has previously been observed in the interaction between *Su(dx)^sp^* and *nd^1^*, although this was less extensive and at a lower penetrance with the *JD* allele (Supplementary Figure S2). At 14 °C, the *Su(dx)^JD^* allele displayed a similar genetic interaction with the *dx^152^* null allele to that previously described for the *Su(dx)^sp^* allele. At this low temperature, *Su(dx)^JD^* wings appear to be wild-type, while *dx^152^* wings have small deltas where the longitudinal veins meet the wing margin (Figure 2H,I). The double homozygous combination of *dx^152^* with *Su(dx)^JD^* results in increased numbers of vein deltas per wing, which are also increased in severity, together with additional vein thickening and cross vein spurs, hence *Su(dx)^JD^* acts as an enhancer of *dx* at this low temperature (Figure 2J,K). At 30 °C, *Su(dx)^JD^* had a similar, recessive, temperature-dependent wing vein loss phenotype to other *Su(dx)* alleles (Figure 1L,M,Q), a phenotype indicative of a Notch gain of function, although the null allele had a lower penetrance for this phenotype than the *sp* allele. *Su(dx)^JD^* failed to complement *Su(dx)^sp^* and *Su(dx)^56^* for this phenotype (Figure 1Q) and, furthermore, the wing phenotypes of *Su(dx)^JD^* were rescued by a single copy of a genomic rescue construct carrying the WT *Su(dx)* gene (Figure 1Q). The *dx* mutation also rescued the *Su(dx)^JD^* wing phenotypes (Figure 1N,Q), as it does with the other alleles [12]. The latter combination, at 25 °C, also resulted in extra leg joints, indicative of increased Notch activity (Figure 1O,P,R), an interaction previously observed with the *Su(dx)^sp^* allele [14]. Unlike with *Su(dx)^sp^*, the null allele also showed a small percentage of legs with extra joints in a WT context, a phenotype which was rescued by the genomic *Su(dx)* rescue construct (Figure 1R). We conclude, therefore, that *Su(dx)^JD^* is a defined null of the *Su(dx)* gene with adult wing and leg phenotypes that are qualitatively similar to those of previously described *Su(dx)* alleles. 

### 3.2. Allele-Specific Differences of Su(dx) Mutant Cyst Packaging Phenotypes during Drosophila Oogenesis

Previous work has suggested that *Su(dx)* is involved in follicle cell production during oogenesis since it was found to be a dominant enhancer of the *daughterless* mutant phenotypes affecting egg chamber separation [25]. However, the consequences of disrupting *Su(dx)*’s function alone in oogenesis have not been investigated. *Su(dx)* was found, by in situ hybridisation, to be expressed in a regulated manner in the ovary. A band of high expression was observed across the region 2a/b of the germarium, which includes the somatic stem cells and follicle cell precursors (Figure 2A–C). *Su(dx)* expression is low in the egg chambers emerging from the posterior of the germarium, but its expression is again up-regulated in stage 6 egg chambers when Notch is active to control the exit from mitosis. All *Su(dx)* alleles tested displayed an egg chamber packaging phenotype; however, there were qualitative differences between the null allele and other tested alleles. *Su(dx)^sp^* and *Su(dx)^sp^/Su(dx)^56^* displayed multi-follicle egg chamber phenotypes, in which a separation between adjacent egg chambers had not occurred, or the separation between egg chambers comprised of a bilayer of cells without an intervening stalk (Figure 2D,E,J–L,P). In both cases, the requisite number of polar cell clusters per egg chamber were found (Figure 2K), i.e., polar cells are normally found in pairs at the anterior and posterior ends of the egg chamber and so, if two egg chambers are unseparated, we expect four pairs to be present [26]. These phenotypes were rarely seen in 3- or 6-day-old adult female ovaries but the frequency of affected egg chambers increased in the ovaries of 9-day-old adults (Figure 2P). These phenotypes were rescued by the presence of a single copy of the WT genomic rescue construct (Figure 2F,P). Even when egg chambers were properly separated, *Su(dx)^sp^* alleles also displayed shorter stalks, with fewer cells than normal located between the egg chambers (Figure 2N,O,Q). Contrary to our expectation, these phenotypes have previously been linked to reduced Notch activity. Consistent with the latter, we found that the *sp/56* mutant phenotypes were enhanced by the loss of one copy of the *Notch* gene, and the phenotypes already appeared in 6-day-old female ovaries in this combination (Figure 2M,P). *Su(dx)^sp^* also displayed an increased number of FasIII-surrounded 16-cell cysts in the germarium, fewer egg chambers and an increase in the stage of the most recent egg chamber to exit from the posterior of the germarium (Figure 2G–I). Together, these phenotypes suggest a problem with supply of follicle cells, which leads to either a failure to separate adjacent egg chambers or decreases the production rate of correctly packaged egg chambers. 

**Figure 3:**
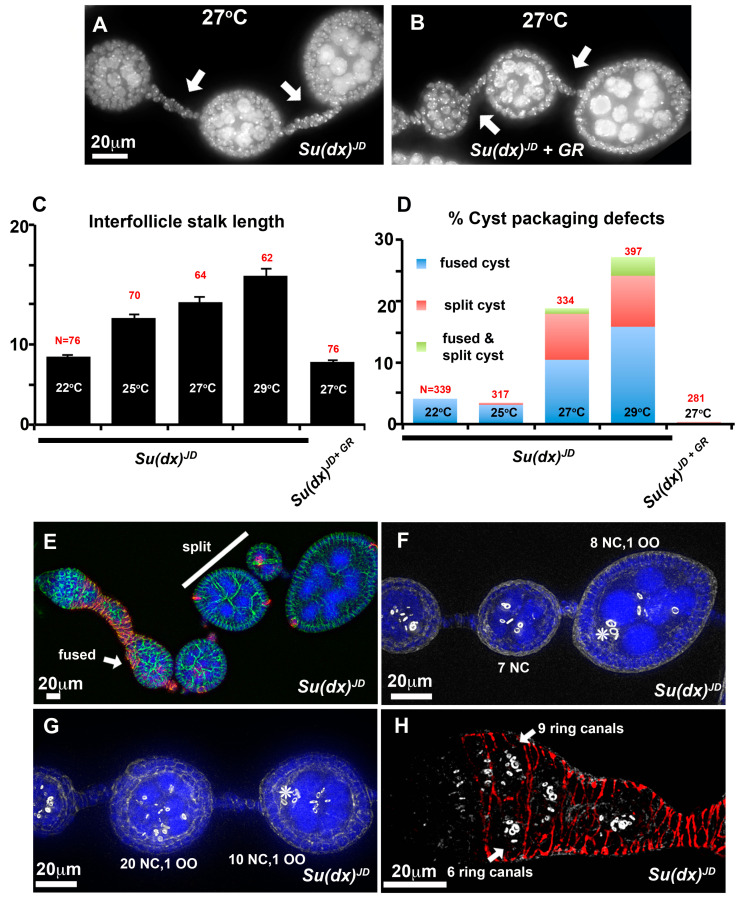
Ovary phenotypes of *Su(dx)^JD^*. (**A**) At 27 °C, *Su(dx)^JD^* has increased stalk length (arrows). (**B**) The *Su(dx)^JD^* stalk length phenotype is reduced by the presence of a genomic rescue (GR) construct of WT *Su(dx)*. (**C**,**D**) Quantification of stalk length (**C**) and cyst packaging phenotypes (**D**) at 14 days post eclosion. Genotypes, temperature and the number of ovarioles scored are indicated. The mean stalk lengths in (**C**) are calculated as the combined mean of stalks located anterior and posterior to stage 4 egg chambers. (**E**–**H**) Examples of cyst packaging phenotypes of two-week-old flies, homozygous for the *Su(dx)^JD^* allele. (**E**) An ovariole containing a split cyst (white bar) in which the full complement of germline cells is split between two egg chambers and a fused cyst (arrow) containing more than the normal 16-cell germline cells. (FasIII, red; anti-Discs large, green; and DAPI, blue). (**F**) Example of a split cyst stained with phalloidin (greyscale) to mark ring canals. Numbers of nurse cells (NC) and oocyte (OO) are indicated. (**G**) Fused/split cysts in which the total complement of 32 germline cells is distributed incorrectly across two adjacent egg chambers, each with one oocyte (OO). * indicates locations of oocyte in F and G. (**H**) Germarium stained with anti FasIII (red) and phalloidin (greyscale), arrows indicate a split cyst already present in region 3 of the germarium.

Unlike in the wing and leg, the ovary phenotypes of the *Su(dx)^JD^* allele markedly differed from the *Su(dx)^sp^* allele, as the null allele flies displayed significantly longer stalks than the wild type, and this phenotype was also rescued by the *Su(dx)* wild-type genomic rescue construct (Figure 3A–C). The long stalk phenotype was temperature-dependent and increased in length at higher temperatures (Figure 3C). The egg chamber phenotypes also differed from those of *Su(dx)^sp^*. Instead of compound and unseparated egg chambers, we observed split cysts in which the normal complement of 16 germline cells were split across two adjacent incomplete follicles or split/fused egg chambers in which some of the germline cells of one egg chamber were split off from one cyst and incorporated into an adjacent chamber (Figure 3D–G). Complete egg chamber fusions that combined two complete cysts into a single egg chamber were also observed (Figure 3E). The splitting of cysts could already be observed within the germarium (Figure 3H). Cyst packaging phenotypes were also rescued by the *Su(dx)* WT genomic rescue construct. 

**Figure 4:**
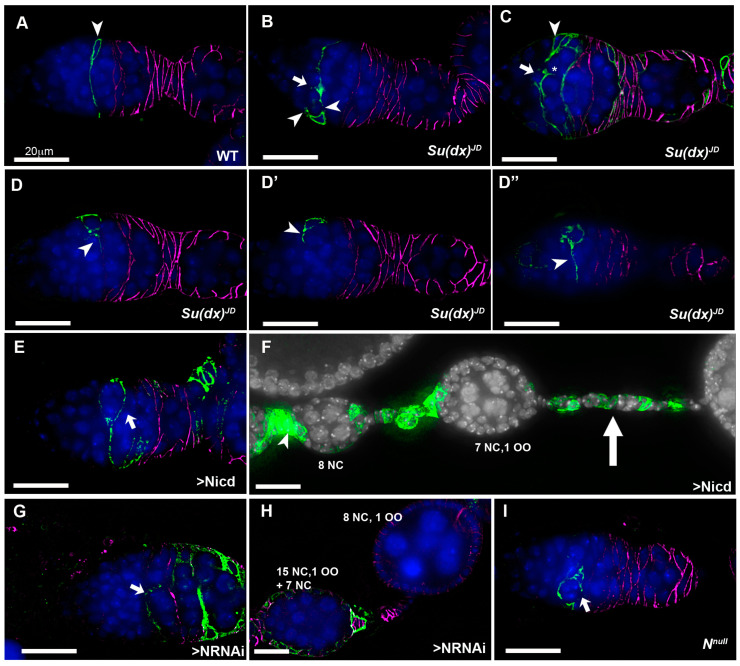
Notch gains and losses of function promote cyst mispackaging in the germarium. (**A**) CD8-GFP (green)-expressing, positively marked WT mitotic clone, originating in a FSC (arrowhead). A long cellular extension spans the width of the germarium. Anti-FasIII marks the follicle cells (purple). (**B**) *Su(dx)^JD^* mitotic clone containing a CD8-GFP-expressing FSC with multiple cell extensions (arrowheads), one of which appears to be in the process of splitting a cyst (arrow). (**C**) A larger marked *Su(dx)^JD^* clone showing a cross-migrating stem cell daughter cell (arrow) from which a cellular process is invading into a cyst (*) Arrowhead marks location of FSC. (**D**–**D”**) A marked *Su(dx)^JD^* FSC has given rise to a number of progeny cells which are each extruding cellular processes in different directions (arrowheads). Three deconvolved sections are shown from a single germarium spaced 60 μm apart. (**E**) A CD8-GFP-marked Notch-ICD-expressing mitotic clone arising from a FSC), which has produced a long cellular process initially in an aberrant direction, and then extending across the germarium, which has enwrapped a partial cyst (arrow). (**F**) Marked clone of cells originating in germarium (arrowhead) expressing Notch ICD generates long stalks separating split cysts (arrow). (**G**) CD8-GFP-marked Notch-RNAi-expressing stem cell with extension that has failed to cross the germarium and instead has partially enwrapped a cyst (arrow). (**H**) A Notch-RNAi-expressing mitotic clone associated with split- and fused-cyst phenotypes. Numbers of nurse cells and oocytes are indicated. (**I**) A *Notch^null^* mutant mitotic clone with cellular process that has failed to extend across the germarium and enwraps a partial cyst (arrow). All scale bars 20 μm.

The follicle cell lineage is maintained by a small number of FasIII-negative follicle stem cells (FSCs) that reside at the region 2a/2b boundary [27]. In this position, the 16-cell germline cysts are forced into a single file and escort cells that wrap the germ line cysts are being replaced by follicle cell progenitors that will eventually form the somatic cell monolayer which encapsulates the egg chambers. In the first step of this replacement, a FSC sends out a long filamentous process that separates successive cysts. FSC division sometimes results in one daughter cell which crosses over the germarium to establish itself posterior to an FSC on the far side [28]. FSCs proliferate to contribute follicle progenitors that surround the cyst as it progresses into region 3 of the germarium. Occasionally a cross-migrating cell moves anterior to the opposite FSC and displaces it from the niche, resulting in stem cell turnover. Notch signalling has been shown to be required for cross-migration and increased Notch activity increases the numbers of cross-migrating cells [28]. By generating homozygous *Su(dx)^JD^* mutant mitotic clones in the stem cells we were able to image mutant cells in the process of cross-migration. In contrast to WT clones, *Su(dx)^JD^* clones appeared disorganised in the extension of these cellular processes, which varied in direction and appeared in some cases to be invading into a cyst, a likely precursory step leading to cyst splitting (Figure 4A–D). Clonal Notch ICD expression (constitutive Notch gain of function) also induced a disorganised over-extension of cell processes causing split-cyst and split/fused-cyst phenotypes with long intervening stalks (Figure 4E,F).

**Figure 5:**
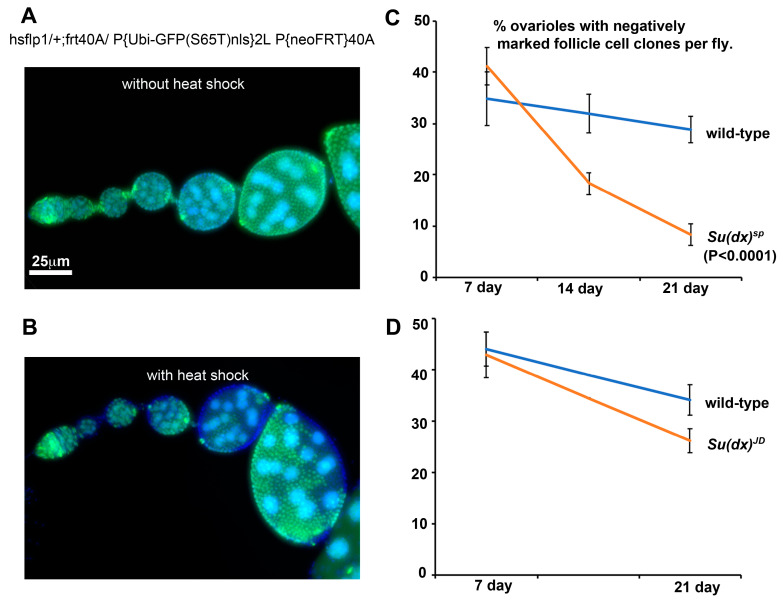
*Su(dx)^sp^* mutation is associated with increased FSC turnover. (**A**) Without the induction of heat shock, mitotic clones are not induced and all cells express a nuclear GFP marker. (**B**) Ovariole after heat shock, showing negatively marked follicle cell clones. (**C**) % of ovarioles exhibiting negatively marked *Su(dx)^sp^* mutant clones shows a greater decline over a 3-week time course than WT clones. This decline is suppressed in a heterozygous *dx^152^* background. (**D**) Turnover of *Su(dx)^JD^* mutant stem cells is not significantly different compared to WT stem cells.

Interestingly, when we expressed Notch RNAi in stem cell clones we also observed the splitting of cysts, but in this case we observed an under-extension of membrane processes which failed to completely cross over to the opposite side of the germarium before enwrapping partial cysts containing less than the normal 16 germline cells (Figure 4G,H). We observed similar outcomes in a *Notch* null mutant clone (Figure 4I). We have not observed split-cyst phenotypes in *Su(dx)^sp^* ovarioles, but we did observe an increased stem cell turnover after a scoring decline in the frequency of permanently marked mitotic clones [29], which was not observed for the *Su(dx)^JD^ allele* (Figure 5). The *Su(dx)^sp^* mutant follicle cell phenotypes may therefore, in part, be due to earlier FSC defects affecting follicle cell supply.

### 3.3. Misregulation of Notch Signalling Levels in the Wing and Ovary

**Figure 6:**
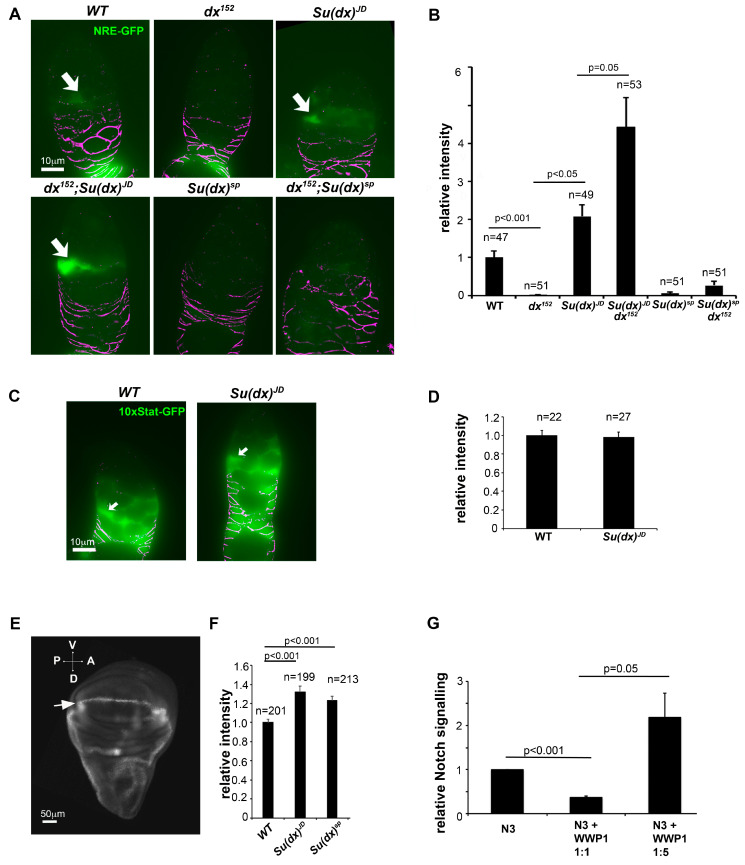
Notch signalling is misregulated by *Su(dx)* alleles in the ovary and wing imaginal discs. (**A**,**B**) Quantification of Notch signalling via the expression of NRE-GFP reporter in cells just anterior to the region of FasIII-expressing cells where the FSCs are located. Images below the graph correspond to the indicated genotypes. Arrows indicate examples of GFP-expressing cells. All images were exposed and processed using same parameters. Anti-FasIII staining in purple. (**C**,**D**) Quantification of JAK-STAT signalling via the expression of the 10× STAT reporter in WT and *Su(dx)^JD^* germaria. Arrows in C indicate positions of FSCs. Error bars in (**B**,**D**) are SEM, no. samples as indicated, *p* values by Mann–Whitney u test. (**E**) Image of wing imaginal disc (posterior left, ventral top) expressing PEST-destabilised superfolder GFP (greyscale). Stripe of Notch activation can be seen along the dorsal/ventral (D/V) boundary (arrow). (**F**) Quantification of NRE fluorescence showing increased GFP expression for both *Su(dx)^sp^* and *Su(dx)^JD^* compared to WT. (**G**) Dosage-dependent regulation of NOTCH3 signalling by human *Su(dx)* homologous protein, WWP1, error bars SEM, *n* = 3, *p* values by *t* test.

The phenotypic characterisation of *Su(dx)* alleles demonstrated that the null allele phenotypes in the wing and leg were consistent with those previously published and indicative of increased Notch activity. A gain of Notch function phenotype was also observed for the null phenotype in the ovary. However, the *Su(dx)^sp^* allele showed ovary phenotypes indicative of a loss of Notch signalling, despite the same allele resulting in a Notch gain of function during wing and leg development. Phenotypic outcomes can be difficult to interpret when multiple processes are affected. Therefore, to observe how different *Su(dx)* mutant alleles affect Notch signalling directly, we investigated the Notch activity marked by fluorescent Notch signal reporters driven by the Notch response element NRE (Figure 6). In the germarium, Notch signalling was detected in cells just anterior to the FasIII-positive cells, in the location of the somatic stem cells, as observed previously [28]. *Su(dx)^sp^* significantly reduced the Notch activity marked by the NRE reporter. In contrast, *Su(dx)^JD^* significantly increased Notch activity (Figure 6A,B). This effect was specific to Notch activity, as *Su(dx)^JD^* did not affect JAK-STAT signalling levels (Figure 6C,D). The latter is a pathway also involved in cyst packaging and egg chamber separation by follicle cells [30]. We tested how the different alleles interacted with a null mutant of *dx* [21]. The *dx^152^* allele alone had reduced Notch activity when measured by this reporter, and there was not any significant rescue of Notch signalling levels when in combination with *Su(dx)^sp^* (Figure 6A,B). However, *Su(dx)^JD^* rescued the Notch signalling loss in a *dx* mutant and this combination had a higher Notch activity than *Su(dx)^JD^* alone (Figure 6A,B). In the wing imaginal discs, both the *Su(dx)^sp^* and *Su(dx)^JD^* alleles increased Notch activation in the dorsal/ventral boundary compared to WT, in agreement with the adult phenotype data that indicated a gain of Notch function in the wings of both mutants (Figure 6E,F). The results further illustrate the complexity of the phenotypic outcomes of the Notch ubiquitin ligase regulators that tune Notch signalling levels up or down in different developmental contexts. We investigated how a human homologue of *Su(dx)*, WWP1, affected human NOTCH3 signalling. Like *Drosophila* Notch, NOTCH3 has a PPXY motif in its intracellular domain which is a recognition motif for the WW domains found in *Su(dx)*-related proteins [31]. We found that WWP1 showed a dosage-dependent regulation of NOTCH3 signalling, decreasing NOTCH3 activity when cotransfected at low ratios but increasing NOTCH3 activity at higher doses of WWP1 transfection (Figure 6G). Thus, both human and *Drosophila Su(dx)* proteins share the potential to act on Notch signalling in either a positive or negative manner. 

## 4. Discussion

Here we report the first defined null allele for *Su(dx)*. We found that *Su(dx)^JD^* behaves similarly to other alleles during wing development, exhibiting temperature-dependent Notch gain of function phenotypes that suppress vein cell fates, acting as a dominant suppressor of *dx* mutant wing phenotypes, and combining with a *dx* null allele to introduce ectopic leg joints, as previously described for other *Su(dx)* alleles [14]. *Su(dx)^JD^* also acted as an enhancer of *dx* mutant wing vein phenotypes when flies raised at 14 °C, a role reversal previously reported for other *Su(dx)* mutant alleles at this low temperature of culture [14]. We further report novel functions of *Su(dx)* during oogenesis, as it regulates cyst packaging into egg chambers and cyst integrity. In this tissue, however, the null allele deviated in its phenotypes from those of other *Su(dx*) alleles. While *Su(dx)^JD^* exhibited the Notch gain of function outcomes of increased stalk lengths and split cysts, the *Su(dx)^sp^* and *Su(dx)^56^* phenotypes reflected their reduced Notch function through short stalks and compound egg chambers. Direct examinations of the Notch signalling levels in vivo, using Notch-specific GFP reporter constructs, were in agreement with the loss or gain of Notch function phenotypic outcomes seen in both the wing and ovary. 

Our observations likely reflect the complexity of the regulatory networks that tune Notch signalling levels up and down through ligand-dependent and independent means, and this context dependency appears likely to extend to human NOTCH regulation. The overall outcome of a particular mutation will therefore depend on the normal balance of these contributions, which differs in different developmental contexts. *Su(dx)^sp^* carries a seven amino acid in-frame mutation in its catalytic HECT domain [8]. Immunostaining showed this allele was expressed and hence it likely retains partial function. The *sp* allele has been characterised as an antimorph as it can compete with WT *Su(dx)* for interactions with Notch. It is probable, therefore, that in the ovary environment, the *Su(dx)*^sp^ protein is still active to sequester Notch from ligand-dependent signalling and this is not compensated for by any increase in ligand-independent activity. Indeed, combinations of *Su(dx)^sp^* and *dx* mutations did not restore Notch signal reporter expression, in contrast to the situation in the wing, where the same mutant combination restored WT wing development. In the latter case, *Su(dx)^sp^* can cause increased Notch activity via a ligand-independent, Dx-dependent mechanism. In contrast, the null allele produces a gain of function in both wing and ovary environments. We found that, in wing development, a *dx* mutation suppresses the gain of function phenotype of the *Su(dx)^JD^* null allele, indicating increased activity through a Dx-dependent route. In the ovary, however, combining *Su(dx)^JD^* and *dx* mutants enhances the increased Notch signalling activity, suggesting that increased ligand-dependent signalling is the dominant contributor to the Notch gain of function phenotype of this combination in the ovary. All *Su(dx)* allele phenotypes were rescued by the introduction of a genomic rescue construct and so the discrepancies between the different allele phenotypes in the ovary cannot be attributed to any second site mutations. A limitation of this study is that we do not understand at the molecular level why the *Su(dx)^sp^* phenotypic outcomes are different in these different tissue contexts. Most likely, subtle differences in the trafficking pathways in which Notch is routed are responsible for these heterogenous outcomes and further detailed studies of Notch localisation are needed.

Our study further uncovered a new role for *Su(dx)* in regulating Notch signalling levels in order to properly package germline cysts into the egg chambers. Cyst packaging is a precisely controlled process [28,32]. After four germ line cell divisions, as it reaches the region 2a/b border, the 16-cell cyst is comprised of 15 nurse cells and a single oocyte, interconnected through cytoplasmic bridges called ring canals. Anterior to region 2a, the cyst is enwrapped by somatic cells called escort cells. As the cyst moves posteriorly, these escort cells are displaced and the cyst becomes surrounded by FasIII-expressing follicle cells. The latter are derived from somatic stem cells located at the 2a/2b border, recognisable from their oval or triangular morphology and lack of FasIII expression [28,33,34]. The number of stem cells present has been a matter of debate in the literature [33,35], but recent work indicates the number is most likely between 2 and 4 active stem cells located either side of the germarium, which is argued to be bilaterally symmetrical [27]. FSC daughter cells can either produce a single cross-migrating cell that moves across the anterior side of the cyst and proliferates to contribute to follicle cells in the anterior half of the egg chamber, or a posterior migrating cell which moves posteriorly and begins enwrapping the posterior face [28,36]. Alternating between these two outcomes is thought to result in each egg chamber being enwrapped by the progeny of two bilaterally located stem cells. Notch signalling has previously been shown to be active in FSCs and acts to promote cross-migration [28]. Using positively labelled clones, we observed that marked stem cells produce a long filamentous process that crosses over the width of the germarium, likely as a precursor to the production of a cross-migrating cell following FSC division. To gain insight into the origin of the split-cyst phenotype, we generated FSC mitotic clones homozygous for the *Su(dx)^JD^* mutant. Germaria were observed with the disorganised extension of cellular processes, which varied in number and direction, arising from stem cells, stem cell progeny and cross-migrating cells. Cytoplasmic processes were observed that appeared to be in the process of invading between cysts. When we expressed the constitutive active Notch intracellular domain in FSC-derived mitotic clones, we also observed a splitting of cysts and similar misregulation of cyst enwrapment. Interestingly, we found that Notch RNAi expression and the stem cell clones of a Notch null mutant also generate split-cyst phenotypes. In this case, this likely results from a loss of cross-migrating cells and incomplete cytoplasmic extensions. Thus, the precise tuning of Notch activity is essential to properly maintain this critical step in egg chamber production.

Notch signalling has previously been shown to be necessary to regulate egg chamber separation subsequent to this initial enwrapment by follicle cells [30,37]. When egg chambers emerge from the posterior of the germaria they are each separated by around six linearly located stalk cells which adjoin the egg chambers through the polar cells that mark the posterior and anterior of each egg chamber. Notch promotes stalk formation and a loss of Notch signalling results in egg chambers that lack an intervening stalk and are separated only by an epithelial bilayer or are compound egg chambers in which multiple cysts are present. We observe similar phenotypes in the ovaries of *Su(dx)^sp^* flies, consistent with their reduced Notch activity. The outcome of this allele results in decreased egg chamber production, reflected in their shorter ovarioles. We also observed a backup of 16-cell germline cysts in the germarium, arrested at the FasIII-enwrapped stage, consistent with an insufficient follicle cell supply. We investigated whether decreased follicle cell production might be reflected by decreased stem cell survival and found that *Su(dx)^sp^*, but not *Su(dx)^JD^*, stem cell mutant clones were lost at a higher frequency compared to wild-type clones. Notch signalling is not thought to be required for stem cell survival. Although previous work found that Mastermind, a key transcriptional coactivator in the Notch pathway, is required for stem cell survival, this was discovered to be due to a non-canonical function of Mastermind downstream of Hedgehog signalling [29]. Previous work, using overexpressed WT and dominant negative *Su(dx)*, has shown that it can target Patched for lysosomal degradation [38]. Since Patched is a negative regulator of Hedgehog, it is possible that *Su(dx)^sp^* mutants act in the stem cells through Ptc accumulation to down-regulate Hedgehog. The overall phenotype of *Su(dx)^sp^* in the ovary may therefore result from the summation of outcomes across these different pathways. 

In conclusion, our results highlight the new roles of *Su(dx)* in oogenesis and illustrate its importance in the precise tuning of Notch activity in different developmental contexts. The core Notch pathway is essentially a simple mechanism, lacking amplification steps or kinase cascades. To be deployed in different developmental contexts Notch requires tunings in its amplitude, duration and spatial pattern in different ways. *Su(dx)* represents one of a multiplicity of regulatory processes that have been uncovered that provide the necessary nuances of Notch regulation that are needed for the proper functioning of such a pleitropic signalling mechanism within the many tissues and cell types in which it functions. Defining a null allele of *Su(dx)* is a step forward in uncovering the full functions of *Su(dx)* in development and tissue homeostasis, but our conclusions may yet be limited by genetic redundancy. It is important, therefore, to address how the functions of *Su(dx)* overlap with other Nedd4 family members in the fly genome to fully understand the complete physiological range of its activity. 

## Data Availability

The data and resources generated during this project are available upon reasonable written request.

## Supplementary Materials

The following supporting information can be downloaded at https://www.mdpi.com/article/10.3390/biom14050522/s1, Figure S1: *Su(dx)* localisation in endosomal compartments; Figure S2: Genetic interaction between *Su(dx)* alleles and the *Notch* allele *nd^1^*.
