# Supplementary material for: The First Defined Null Allele of the Notch Regulator, a Suppressor of Deltex: Uncovering Its Novel Roles in Drosophila melanogaster Oogenesis
Source: Biomolecules. 2024 Apr 26;14(5):522. doi: 10.3390/biom14050522 (PMC11118177; doi:10.3390/biom14050522)
Supplement: Supplementary file 1 [file biomolecules-14-00522-s001.zip › biomolecules-2937112-supplementary.pdf]

Supplemental figure legends

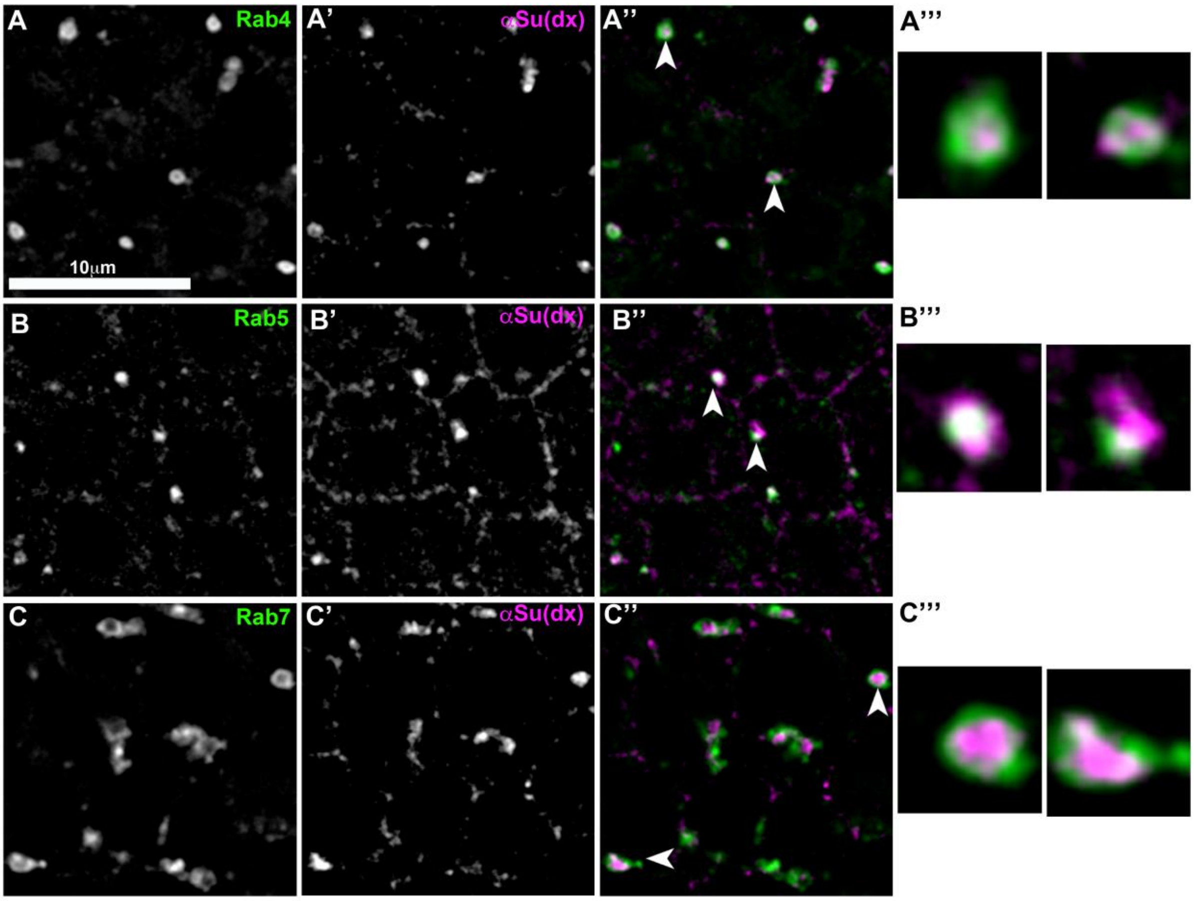

Supplemental Figure S1. Localisation of Su(dx) in the endocytic pathway. Anti Su(dx) (magenta) localisation in organelles marked with endogenously tagged **(A-A′′′)** Rab4-EYFP, **(B-B′′′)** Rab5-EYFP and **(C-C′′′)** Rab7-EYFP (Green). Arrow heads mark endosomes depicted in enlarged images in **A′′′**,**B′′′** and **C′′′**. Immunostaining performed on egg chamber follicle cells.

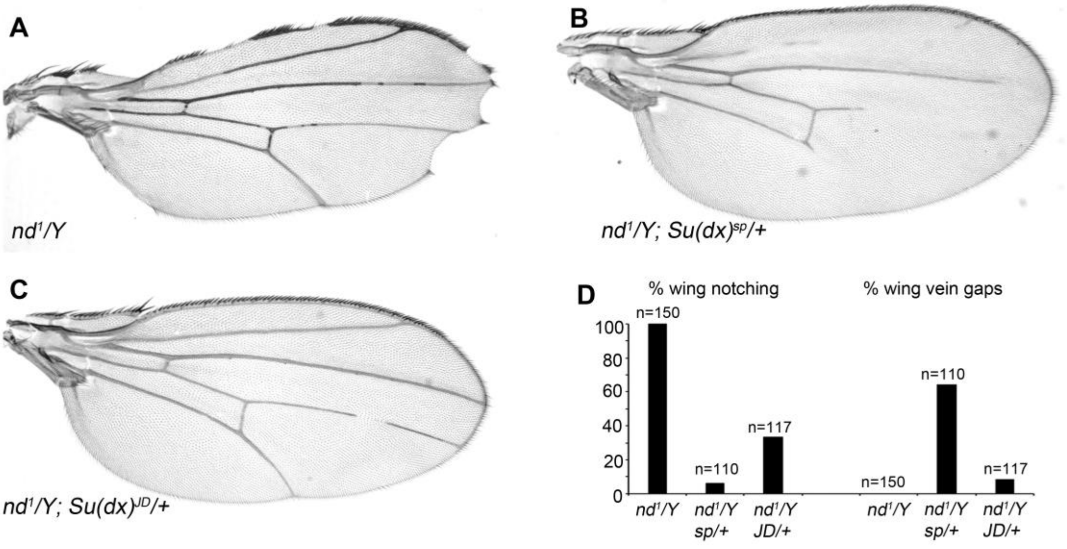

Supplemental Figure S2. Genetic interaction of *Su(dx)* alleles with *Notch* hypomorphic allele *nd¹*. (A) Wing of *nd¹/Y* fly showing extensive notching around the wing margin. **(B)** Heterozygous *Su(dx)ˢᵖ* allele, suppresses the wing margin notching of *nd¹/Y* and introduces strong wing vein gap phenotype. **(C)** Heterozygous *Su(dx)ᴶᴰ* allele, also suppresses the wing margin notching of *nd¹/Y* and introduces wing vein gap phenotype but these are smaller and less penetrant that in the interaction with *Su(dx)ˢᵖ*. **(D)** Quantification of genetic interactions depicted in **A**-**C**. *Su(dx)ᴶᴰ* allele behaves in a qualitatively similar manner to *Su(dx)ˢᵖ* but with a less penetrant outcome.
